# Bile Acids, Liver Cirrhosis, and Extrahepatic Vascular Dysfunction

**DOI:** 10.3389/fphys.2021.718783

**Published:** 2021-07-29

**Authors:** Tilman Sauerbruch, Martin Hennenberg, Jonel Trebicka, Ulrich Beuers

**Affiliations:** ^1^Department of Internal Medicine I, University of Bonn, Bonn, Germany; ^2^Department of Urology I, University Hospital, LMU Munich, Munich, Germany; ^3^Translational Hepatology, Medical Department, University of Frankfurt, Frankfurt, Germany; ^4^Department of Gastroenterology and Hepatology, Amsterdam University Medical Centers, location AMC, Amsterdam, Netherlands

**Keywords:** bile acids, liver cirrhosis, portal hypertension, microbiome, vasodilation

## Abstract

The bile acid pool with its individual bile acids (BA) is modulated in the enterohepatic circulation by the liver as the primary site of synthesis, the motility of the gallbladder and of the intestinal tract, as well as by bacterial enzymes in the intestine. The nuclear receptor farnesoid X receptor (FXR) and Gpbar1 (TGR5) are important set screws in this process. Bile acids have a vasodilatory effect, at least according to *in vitro* studies. The present review examines the question of the extent to which the increase in bile acids in plasma could be responsible for the hyperdynamic circulatory disturbance of liver cirrhosis and whether modulation of the bile acid pool, for example, *via* administration of ursodeoxycholic acid (UDCA) or *via* modulation of the dysbiosis present in liver cirrhosis could influence the hemodynamic disorder of liver cirrhosis. According to our analysis, the evidence for this is limited. Long-term studies on this question are lacking.

## Introduction

**Figure 1:**
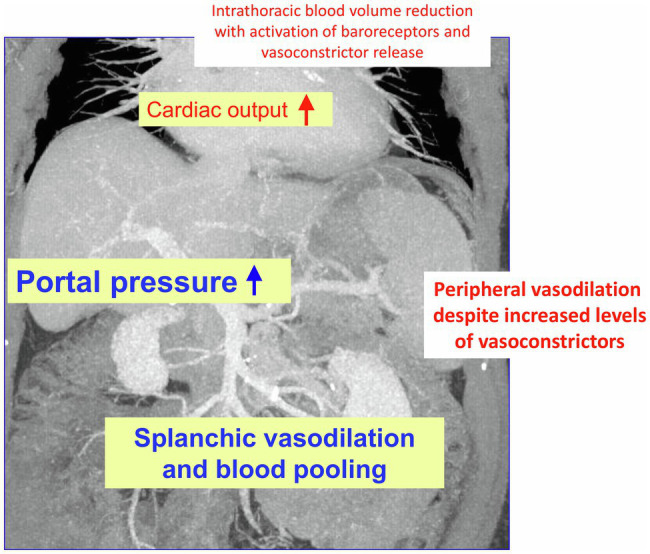
Changes in hemodynamics in liver cirrhosis: As decompensation progresses and portal vein pressure increases, there is initially splanchnic vasodilation with pooling of abdominal blood volume and concomitant reduction in intrathoracic blood volume. Later, peripheral vasodilation is also observed. This results in hyperdynamic circulatory dysfunction with an increase in cardiac output. The compensatory release of vasoconstrictors (partly *via* activation of baroreceptors) cannot completely resolve the hemodynamic dysfunction partly because the vessels have an impaired response to vasoconstrictor stimuli, partly due to formation of vasodilators such as nitric oxide (NO).

In liver cirrhosis, there is a marked change in liver perfusion and extrahepatic hemodynamics. The consequences are severe: formation of collaterals bypassing the liver, as well as splanchnic and peripheral systemic vasodilatation with a hyperdynamic circulation and central hypovolemia (Schrier et al., 1988; Maroto et al., 1994; Blendis and Wong, 2001; Moller et al., 2011). These alterations on the one hand increase portal pressure and on the other hand fuel dysfunction of other organs such as of kidneys (Wong et al., 2020) and lungs (Goldberg and Fallon, 2015). The heart may be involved too, in the complex of hyperdynamic circulatory dysfunction or due to cirrhotic cardiomyopathy. To this, the body counter-reacts with activation of the renin-angiotensin system (Bosch et al., 1980) and release of other vasoconstrictors such as catecholamines or vasopressin (Henriksen et al., 1984; Figure 1). This may lead to a vicious circle supporting generation of ascites, development of a hepatorenal syndrome, and life-threating variceal bleeding (Sola and Gines, 2015). The hemodynamic changes increase with the degree of liver dysfunction, but are not specific to the particular cause of cirrhosis. They can equally be detected in different animal models (Heller et al., 2005; Hennenberg et al., 2009a).

In addition to initial chronic liver inflammation causing alterations in the intrahepatic blood flow, the intestine has become a focus of pathophysiologic consideration in recent years. Dysbiosis and a disturbed intestinal barrier allow the translocation of molecules, microorganisms, or their products into the interior of the body, where they act as inflammatory stimuli, especially within the liver (Fairfield and Schnabl, 2021).

Here, we would like to deal mainly with one group of molecules within these complex systems, namely bile acids (BA) and their possible role in the development of changes in hemodynamics in the context of liver cirrhosis with a focus on the extrahepatic vessels. Primary bile acids are formed in the hepatocytes and then underlie an enterohepatic circulation. Thereby, they are subjected to strong intestinal modification. Thus, chronic liver diseases intervene alongside with the intestinal environment in bile acid composition and their pool (Ridlon et al., 2015; Fickert and Wagner, 2017). Unfortunately, we have only incomplete knowledge about the exact distribution of the various bile acids in different organs and compartments. During the course of liver disease, considerable changes occur. Model calculations attempt to approximate this changing process (Voronova et al., 2020). Bile acids have distinct functions. They are breakdown products of cholesterol and they are detergents that play an important role in fat digestion and vitamin intake. Furthermore, it has been shown in recent decades that bile acids are hormones, ligands for receptors, and transcription factors (Hofmann and Hagey, 2014). Bile acids also affect vascular function. This we would like to discuss in the context of liver cirrhosis.

## Hemodynamic Alterations in Liver Cirrhosis

Roberto Groszman and his group were one of the first to show that there is a paradox in liver cirrhosis: an increased vasoconstriction of the intrahepatic microcirculation with a concomitant vasodilation outside the liver (Iwakiri and Groszmann, 2007) concerning first splanchnic and then peripheral systemic vessels. They and others attributed this quite substantially to decreased formation of the vasodilator nitric oxide (NO) within the liver (Iwakiri et al., 2014) and increased NO generation outside the liver.

Indeed, one finds an increasing excretion of nitrite/nitrate as a surrogate marker for NO formation in humans parallel to worsening of liver function (Heller et al., 1999a). By contrast, stimulation of endothelial NO synthesis using an enhancer of endothelial NO synthase (eNOS) transcription lowers intrahepatic resistance and *in situ* perfusion pressure in cirrhotic rat livers (Biecker et al., 2008). Similarly, the beneficial effect of atorvastatin in liver cirrhosis is associated with increased activity of eNOS (Trebicka et al., 2007). These findings underscore the hypothesis of impaired intrahepatic NO formation as a cause of intrahepatic resistance augmentation in addition to structural remodeling of the liver. Cells involved in the regulation of the intrahepatic vascular bed include endothelial cells, smooth muscle cells, and hepatic stellate cells (HSCs, a form of intrahepatic pericytes). It is assumed that sinusoidal endothelial cells change their phenotype on the way to cirrhosis and produce less NO causing a shortage of vasodilating stimuli on smooth muscle cells and HSC (Iwakiri et al., 2014).

Regarding extrahepatic vasodilation (Figure 1), a number of mechanisms are discussed, originating from different structures of the vessels – such as adventitia, smooth muscle, and endothelium – and its neuronal supply. These may influence each other paracrinally or respond to systemic and nerval stimuli.

The essential role of NO-mediated splanchnic and systemic vasodilation and hyporeactivity to vasoconstrictors in liver cirrhosis was highlighted in the 1990s using animal models (Wiest and Groszmann, 2002). NO is produced *via* different synthases (endothelial, eNOS; neuronal NO synthase, nNOS; and inducible NO synthase, iNOS). According to the classical hypothesis of Vallance and Moncada (Bhagat and Vallance, 1996; Vallance et al., 1997), inflammatory stimuli (e.g., endotoxin) were thought to upregulate iNOS in vascular smooth muscle cells in liver cirrhosis – at least for initiation of vasodilatation. Animal experiments, however, pointed to a much more important role of eNOS (Wiest et al., 1999b; Wiest and Groszmann, 2002). According to these findings, NO is formed in the vascular endothelium and causes cGMP-mediated relaxation of the adjacent smooth muscle cell leading to vascular dilatation. The upregulation of eNOS seems to depend more on the degree of portal hypertension and less on the extent of chronic inflammation. Shear stress is hypothesized as a major pathomechanism, but other not fully elucidated molecular mechanisms are also suggested. eNOS may also be upregulated by bacterial translocation and proinflammatory cytokines (Wiest et al., 1999a). Despite these findings, there is indirect evidence, that iNOS also contributes to peripheral vasodilation in humans with decompensated liver cirrhosis (Ferguson et al., 2006). Finally, increased expression of neuronal NOS was also found, suggesting additional nerve-mediated NO formation. However, findings in this direction are sparse (Moll-Kaufmann et al., 1998). This pathophysiological role of NO in vasodilation unraveled in experimental animals with liver cirrhosis has also been confirmed in humans to a certain degree (Albillos et al., 1995; Battista et al., 1997; Heller et al., 1999a; Helmy et al., 2003).

In liver cirrhosis, a well-characterized activation of the renin-angiotensin-aldosterone system (RAAS) occurs, as mentioned above, in part as response to systemic vasodilation, especially in patients with ascites (Bosch et al., 1980; Helmy et al., 2000; Schepke et al., 2001b). Notably, both in animal models and in humans, an upregulation of ACE2 and of the Mas receptor in the splanchnic vessels result in locally increased angiotensin (1–7) levels – derived from the increased circulating angiotensin II – generating NO mediated *via* the MAS receptor. Thus, the alternate arm of RAAS in patients with liver cirrhosis can cause vasodilatation (Grace et al., 2013).

Nitric oxide is certainly not the only factor driving vasodilation in liver cirrhosis. Many other vasodilating molecules, such as adrenomedullin (mediated by inflammatory stimuli) carbon monoxide (CO), formed by heme oxygenase-1 (endothelial), prostacyclins (PGI_2_), endothelial derived epoxyeicosatrienoic (EET) acids, cannabinoids (such as anandamide), or glucagon show elevated plasma levels in liver cirrhosis (Hennenberg et al., 2008; Di Pascoli et al., 2017).

**Figure 2:**
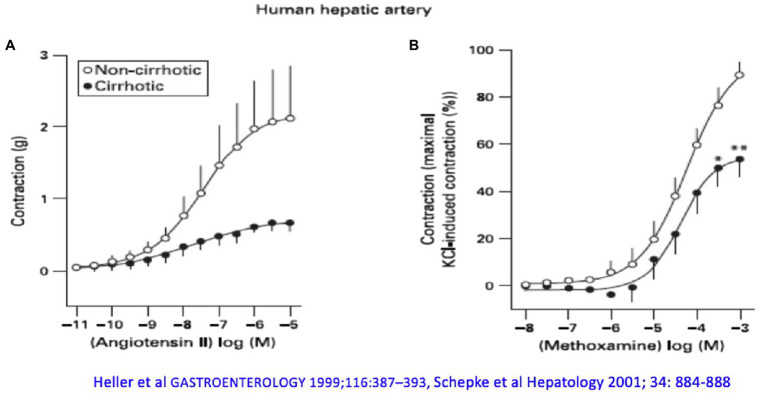
The arterial hepatic vessels of patients who underwent liver transplantation show an impaired vasoconstrictor response compared with the vessels from donors: **(A)** to angiotensin II and **(B)** to methoxamine. Modified according to Heller et al. (1999b) and Schepke et al. (2001a).

However, increased formation of vasodilators is not the whole explanation for altered hemodynamics in liver cirrhosis. Human hepatic arteries without endothelium from patients with liver cirrhosis obtained during liver transplantation respond significantly worse to vasoconstrictors (α_1_- and α_2_-adrenergic agonists) compared with corresponding vessels from donors, even after pharmacological blockade of NOS (Figure 2). Receptor-independent membrane depolarization with potassium chloride, direct stimulation of the phospholipase C/inositol-1,4,5-trisphosphate (PLC/IP_3_) axis, or the G protein pathways led to a similar contraction, so that the contractile proteins were apparently not affected to a major extent by the cirrhotic status (Heller et al., 1999b; Schepke et al., 2001a). Arterial hypocontractility to α_1_-adrenergic agonists can also be consistently demonstrated in animal experiments of liver cirrhosis (Hennenberg et al., 2009b), even after removal of the endothelium and blockade of NO synthase. Here, a defective activation of Rho-kinase (ROCK) has been found (Hennenberg et al., 2006), possibly caused by an increased binding of GRK-2/β-arrestin-2 to the AT_1_ receptor (Hennenberg et al., 2007). Dysregulation of the neurotransmitter NPY, which enhances α_1_-adrenergic vasoconstriction, is also noted in the cirrhotic rat (Moleda et al., 2011). Decreased transcription of vasoconstrictor receptors does not seem to play a role (Neef et al., 2003).

The progressive hepatic impairment with alteration of hemodynamics is additionally accompanied by increasing systemic inflammation (Praktiknjo et al., 2020a), which may be complicated by an acute event leading to rapid failure of one or more organs, a situation now coined acute on chronic liver failure (ACLF; Moreau et al., 2013; Arroyo et al., 2021). During deterioration of disease phenotypic changes of the immune cells occur (Weiss et al., 2020) together with an activation of the inflammasomes (Praktiknjo et al., 2020b; Monteiro et al., 2021) and increases of different cytokines, e.g., Il-6, Il-17, IL-1: a condition we know from subclinical inflammation and sepsis due to other causes (Arina and Singer, 2021). Recent research has increasingly focused on the gut as a major source and trigger of this stimulation of the immune system. Intestinal dysbiosis (a change of the composition of the microorganisms and their diversity) and disturbance of the gut barrier to microorganisms and their metabolites are discussed as major causes (Trebicka et al., 2021). The vasodilatory co-reaction of the vessels in such an inflammatory state has been known for a long time, and the question arises to what extent patient’s hemodynamics is altered by inflammatory changes coming from the gut.

Since bile acids (i) are in constant exchange between the hepatic, biliary, intestinal and – to a much lesser degree- plasma compartments *via* the enterohepatic circulation and spill-over into the systematic circulation; (ii) are modified *via* intestinal microorganisms; (iii) interact with immune cells; and (iv) also influence vascular contraction, it is interesting to consider a role of these molecules in the dynamics of the systemic circulation.

## Physiology of Vasoconstriction and Vasodilatation

Vasocontraction and relaxation essentially contribute to dynamic changes in vessel diameter. It is largely based on dynamic phosphorylation and dephosphorylation of contractile proteins in smooth muscle cells. This in turn depends on calcium homeostasis in the cell, controlled on the one hand by release from intracellular calcium stores, and on the other hand by calcium channels of the plasma membrane, both taking part in a cascade of intracellular signaling toward phosphorylation of myosin light chains (MLC; Hennenberg et al., 2008). This cascade is activated by binding of vasoconstrictors, including catecholamines or vasoactive peptides such as angiotensin II or endothelin-1, to their cognate, G protein coupled receptors. Calcium-independent mechanisms are activated in parallel, which essentially act *via* modulation of MLC phosphorylation as well. Essentials are the RhoA/ROCK pathway and the PLC-DAG/PKC pathway (Hennenberg et al., 2008; Touyz et al., 2018). Thus, ROCK activation blocks MLC phosphatase and therewith facilitates contraction which needs phosphorylation of the MLC. Meanwhile, other non-RhoA GTPases are also discussed in smooth muscle cell regulation, such as Rac1 (Li et al., 2020). Important vasodilatory pathways work *via* formation of the two cyclic nucleotides cGMP and cAMP in smooth muscle cells. Both pathways involve activation of MLC phosphatase and decreases in cytosolic calcium concentrations, with the latter being based on regulation of voltage-gated calcium channels, calcium-dependent (BK_Ca_), ATP-sensitive (K_ATP_), and other potassium channels, sarco−/endoplasmic reticulum Ca^2+^ ATPase, as well as other targets. cGMP formation results from paracrine guanylyl cyclase activation by NO, following either acetylcholine- or shear stress-induced eNOS activation in endothelial cells or its release from nitrergic neurotransmisssion and its immediate dissociation into smooth muscle cells. Formation of cAMP and cAMP-mediated vasodilation are induced by prostaglandins and β-adrenoceptors, which activate corresponding receptors located on vascular smooth muscle cells, thereby activating adenylyl cyclase.

Chronic conditions such as subclinical inflammation or stress can probably alter the phenotype and plasticity of smooth muscle cells *via* differential expression of contractile proteins as well as proteins involved in various above mentioned signaling cascades and calcium regulation. However, there are no good studies on the phenotypic change of the vascular smooth muscle cell for the situation of liver cirrhosis.

## Bile Acid Pool, Bile Acid Composition and Their Regulation

**Figure 3:**
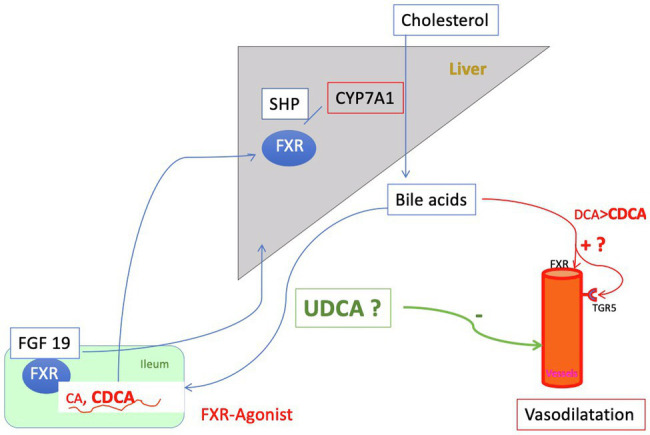
Regulation of the bile acid pool: The primary bile acids (BA) cholic acid (CA) and chenodesoxycholic acid (CDCA), newly formed by the liver and conjugated there are secreted into the intestine and mostly reabsorbed by active transport in the ileum. Around 20% of the primary BA are metabolized in the intestine by bacterial enzyme to secondary bile acids [deoxycholic acid (DCA) and small amounts of lithocholic acid (LCA)], predominantly by loss into the colon. Here, the secondary bile acids are passively absorbed and returned to the liver. Only 5% of the bile acid pool is lost daily *via* the feces. This process of enterohepatic circulation is regulated by gallbladder and intestinal motility and nuclear farnesoid X receptor (FXR). FXR is expressed in the enterocyte of the ileum as well as in the liver. Its action is mediated on the one hand *via* small heterodimer partner (SHP) and on the other hand hormonally *via* Fibroblast growth factor (FGF) 19, which suppresses bile acid formation. Thus, the process of enterohepatic circulation is regulated to a large extent by gallbladder and intestinal motility and FXR. In liver cirrhosis, there is an overall reduction in the bile acid pool with a relative increase in CDCA. At the same time, the concentration of conjugated bile acids in the systemic circulation increases sharply, especially that of CDCA. CDCA is an activating ligand for FXR and also for TGR5 and in this way releases NO, i.e., may cause vasodilation. At the same time, however, activation of FXR has beneficial effects (e.g., downregulation of inflammation). Increased concentrations of bile acids in the plasma compartment found in liver cirrhosis might cause vasodilation either directly or *via* FXR and the G protein-coupled bile acid receptor 1 or TGR5 (see Figure 6). Ursodeoxycholic acid (UDCA) is almost inert for FXR and TGR5 and is hepatoprotective. Whether it is useful to modulate the bile acid pool in liver cirrhosis by administration of UDCA for this reason is discussed in the text.

The two primary BA, cholic acid (CA), and chenodeoxycholic acid (CDCA), are formed predominantly in the pericentral hepatocytes over several steps from cholesterol. In this process, cholesterol 7-alpha-hydroxylase (CYP7A1) is the rate-determining enzyme (classical pathway). After conjugation with glycine or taurine in the peroxisomes, the bile acids are actively transported into the bile canaliculus (Fickert and Wagner, 2017). Here, they form micelles with phosphatidylcholine and also cholesterol and enter *via* the bile ducts the duodenum. In the intestine, BA are important for fat digestion. Furthermore, as a degradation product of cholesterol, they have a pivotal function in the cholesterol homeostasis of the body (Hofmann, 2009). Re-uptake of BA occurs *via* apical and basolateral transporters of the ileal enterocytes (Figure 3). Around one fifth of BA is not absorbed in the ileum and passes into the colon. Here, deconjugation and dehydroxylation to secondary bile acids occur *via* bacterial intestinal enzymes. Most of these BA are passively reabsorbed by the colon and enter the circulating bile acid pool. Only a small percentage (around 5%) of the whole BA pool leaves the body every day *via* the stool, helping thereby to determine the cholesterol balance (Chiang and Ferrell, 2020). Thus, the intestine with its microbiota plays an important role in the modification and subsequent composition of the bile acid pool. After return to the liver *via* the portal venous blood BA are actively reabsorbed from the sinusoidal blood *via* basolateral transporters on the hepatocytes and, after passage through the liver cell, are reintroduced into the biliary ductal system. In this circuit, tuning of synthesis and transport occurs through nuclear receptors – primarily the nuclear farnesoid X receptor (FXR) – and hormones, mainly through fibroblast growth factor (FGF) 15 (rodent)/19 (human). FXR primarily reduces intestinal bile acid uptake and bile acid synthesis in the liver. Furthermore, it enhances bile acid biotransformation and export into bile (and back into blood when biliary secretion is impaired). Ligands for FXR are, in descending order CDCA > LCA = DCA > CA. FGF 19 is FXR-dependently formed in the ileocyte under physiological conditions and secreted into venous mesenteric blood. Reaching the liver *via* the portal vein, FGF19 binds to the hepatocellular FGF receptor 4 (FGFR4)/ß-klotho complex and suppresses bile acid formation at the level of CYP7A1, the key enzyme for bile acid synthesis. At the same time, FGF19 leads to a dilatation of the gall bladder (Kliewer and Mangelsdorf, 2015). In healthy individuals, the bile acid pool modulated in this way contains predominantly CA (around 40%), CDCA (around 40%), and DCA (around 20%). The size of the pool is about 2 to 5 g. The concentration of total bile acids in the biliary system is 20–40 mmol/L, in the gall bladder 50–200 mmol/L, in the small intestine 20–50 mmol/L, in the portal blood 20–50 μmol/L, and in the liver <50 μmol/L (Kliewer and Mangelsdorf, 2015; Fickert and Wagner, 2017). A spill over into the systemic circulation leads to a plasma concentration of total bile acids of about 2–8 μmol/L in the fasted state (Schalm et al., 1978; Hofmann, 1994). This situation is considerably changed in hepatic disease, especially in liver cirrhosis.

## Changes of Bile Acids in Liver Cirrhosis

**Figure 4:**
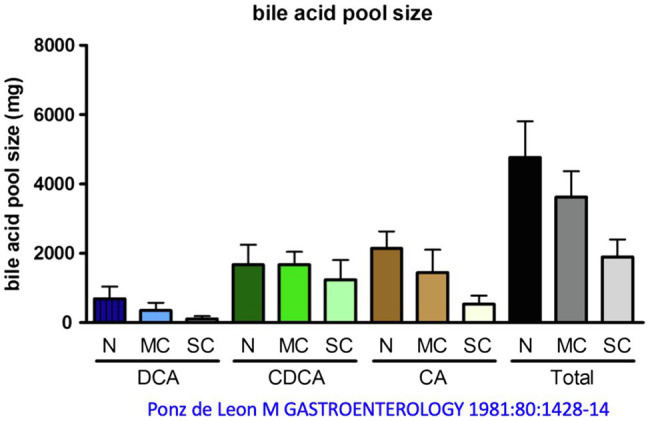
Change in bile acid pool in patients with mild cirrhosis (MC) and severe cirrhosis (SC) compared with normal subjects (N). DCA, deoxycholic acid; CDCA, chenodeoxycholic acid; CA, cholic acid. Modified by data from Ponz de Leon et al. (1981).

The bile acid pool of a healthy 70 kg person, which circulates about 5–10 times per day enterohepatically, ranges between 2 and 5 g, as mentioned. Distributed in this pool are the primary bile acids cholic acid (CA, around 40%), CDCA (around 40%), and the secondary bile acid DCA (around 20%), see above. The secondary bile acid lithocholic acid (LCA) and the tertiary bile acid ursodeoxycholic acid (UDCA) account for only a few percent. About one-third of these bile acids are taurine conjugates and two-thirds are glycine conjugates (Carey and Duane, 1994; Hofmann, 1994; Chiang and Ferrell, 2020). In cirrhosis, the bile acid pool decreases as a function of the extent of decompensation, according to one report (Ponz de Leon et al., 1981) from just under 5 g (controls) to 4.6 g [mild cirrhosis (MC)] and just under 2 g in severe decompensated cirrhosis. This decrease affects quite substantially CA and DCA, but hardly CDCA, i.e., there is a relative accumulation of CDCA in liver cirrhosis (Vlahcevic et al., 1981; Figure 4). Conjugated CDCA is the major endogenous ligand for FXR. Reduction of CA and DCA may be in part caused *via* increased FXR-mediated formation of FGF 19 in the ileum (Brandl et al., 2018). FGF 19 inhibits bile acid synthesis in the liver *via* binding to the FGFR4 and inhibition of CYP7A1 or directly *via* induction of small heterodimer partner (SHP) expression in the hepatocyte.

The ratio of bile acid concentration of portal venous to peripheral venous blood is about 7–10: 1 in healthy individuals (Angelin et al., 1982), with concentrations increasing by a factor of 2–3 in postprandial portal venous blood (Angelin et al., 1982). The fluctuating concentrations in venous peripheral blood are much less pronounced (LaRusso et al., 1974; Schalm et al., 1978; Angelin et al., 1982). In contrast, in liver cirrhosis, the total bile acid concentration in the peripheral venous blood corresponds to the concentration in the portal vein or may even be higher, partly due to shunting (Ohkubo et al., 1984). In blood, bile acids are tightly bound to serum albumin and lipoproteins (Rudman and Kendall, 1957; Kramer et al., 1979; Roda et al., 1982; Ceryak et al., 1993), hydrophobic bile acids predominantly to albumin and hydrophilic bile acids also to lipoprotein particles, especially HDL and LDL.

The serum concentration of total bile acids increases (especially in alcoholic cirrhosis; Ciocan et al., 2018) with the degree of decompensation of liver cirrhosis and is prognostic for survival (Mannes et al., 1986; Wang et al., 2016). The same holds true for hemodynamic parameters. In parallel, the amount of serum bile acids correlates with the degree of portal hypertension (Horvatits et al., 2017a) and hepatopulmonary syndrome (Horvatits et al., 2017b). These observations raise the question of a causal role of bile acids in the pathogenesis of impaired hemodynamics in liver cirrhosis.

The vast majority of bile acids in peripheral plasma of patients with alcoholic cirrhosis are conjugated with a shift toward taurin-conjugates (nearly 1:1 ratio of glycine- to taurine-conjugates; Brandl et al., 2018; Ciocan et al., 2018; Trefflich et al., 2019). Similar to the bile acid pool, as disease progresses toward liver cirrhosis, the relative proportion of CDCA increases in serum. This is especially true for severe alcoholic cirrhosis with alcoholic hepatitis (Brandl et al., 2018; Ciocan et al., 2018) and less pronounced in hepatitis B-virus induced cirrhosis, primary biliary cholangitis (PBC) or primary sclerosing cholangitis (PSC; Azer et al., 1996; Dilger et al., 2012; Wang et al., 2016; Ciocan et al., 2018; Liu et al., 2018; Chen et al., 2020).

Driven by the increase of intrahepatic resistance to portal venous outflow with the development of portal hypertension and due to the remodeling of the intrahepatic vascular architecture, intra-, and extrahepatic shunts develop. Recent computed tomography-based studies show that more than half of the patients with liver cirrhosis have spontaneous portosystemic shunts, partially large. In Child-C cirrhosis, the percentage rises to above 70 (Simon-Talero et al., 2018). In patients with liver cirrhosis who underwent shunt surgery, it has been shown that this leads to a permanent increase in serum bile acids (Poupon et al., 1976; Capocaccia et al., 1981). These findings suggest that altered splanchnic hemodynamics is a major cause of the elevated serum bile acid levels. In an older, very elegant paper, Ohkubo et al. (1984) showed in patients with compensated cirrhosis that there is a highly significant correlation between portal venous shunt index as a measure of spontaneous shunting and peripheral venous bile acid concentration. In dogs, the creation of a portocaval end-to-side shunt leads to a marked increase in bile acid, which can be prevented by arterialization of the truncated portal vein stump (Horak et al., 1975). These findings suggest that the increase in serum bile acids of cirrhotics is much more determined by disturbances of the hepatic blood flow, i.e., perfusion of the sinusoids, than by reduced extraction of the hepatocytes. The authors assume that the induction of the partial and permanent bypass of hepatic extraction is not caused by a vasoactive effect of bile acids. However, whether bile acids later contribute to a vicious circle in bypassing the liver is unclear.

To summarize, the circulating bile acid pool decreases in liver cirrhosis, showing a relative increase in CDCA (especially in alcoholic cirrhosis) and a significant spillover into the systemic circulation, depending on the extent of decompensation of cirrhosis, so that serum concentrations of total bile acids approach those in portal blood. Here, the proportion of unconjugated bile acids decreases and the relative proportion of taurine conjugates increases.

## Direct Effects of Bile Acids on the Vasculature

There is the clinical – but systematically poorly studied – observation that patients with protracted cholestasis and jaundice have low systemic blood pressure. In rats or mice bile duct ligation leads to systemic vasodilation and hypotension (Bomzon and Ljubuncic, 1995; Heller et al., 2005; Hennenberg et al., 2006, 2007, 2009a). Therefore, some authors speculated that a direct vasodilatory effect of bile acids might be responsible. Indeed, Pak and coworkers (Pak and Lee, 1993) showed that infusion of TCDCA and TDCA in particular increased mesenteric blood flow, decreased systemic blood pressure, and dilated precontracted mesenteric vascular rings in cirrhotic rats and – more so – in controls. In the following, we would like to further discuss the vasodilatory effect of bile acids.

**Figure 5:**
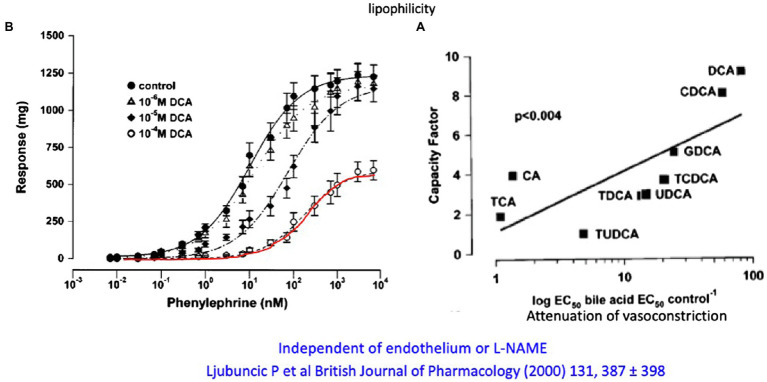
**(A)** Attenuation of the *in vitro* contractile response to phenylephrine of aortic rings from rats by different concentrations of DCA. **(B)** Relation of the lipophilicity of different conjugated and unconjugated and their attenuation of the vasoconstrictive response of aortic rings to phenylephrine (modified according to Ljubuncic et al., 2000).

A very careful systematic work (Ljubuncic et al., 2000) investigated the effect of different primary and secondary, taurine- and glycine-conjugated bile acids on phenylephrine- and methoxamine-induced contraction on vascular rings of the abdominal aorta in the rat as well as their possible vasodilatory effect on precontracted aortic rings. Unconjugated CDCA and even more pronounced CDCA but not CA at micromolar to millimolar concentrations were shown to significantly reduce maximal vascular contraction. With the exception of CA, all conjugates (DCA, CDCA, and UDCA) also increased the EC50 value for α_1_-adrenergic ligands but not the maximal response (Figure 5). There was a linear relationship between the reduction in contractile response and the relative fat solubility of bile acids. Unconjugated DCA and CDCA had the strongest effect, followed by GDCA and TCDCA. The vasorelaxant effect of DCA was not affected by removal of endothelium or blockade of NO formation using L-NAME. The authors speculated that lipophilic bile acids affect the affinity of adrenoceptors by increasing lipid peroxidation (and thus altering the membrane micromilieu at the receptor), possibly also affecting the fluidity of the membrane.

In contrast, another group (Khurana et al., 2005) found in *in vitro* studies using pre-contracted rat and mouse thoracic aortas that the vasodilating effect of taurine conjugated DCA (up to 1 mmol/L) was nearly abolished by blocking NO synthesis (by removing endothelium) or by knockout of the muscarinic M3 receptor. They concluded that the relaxing effect of this bile acid is mediated *via* the endothelial M3 receptor and release of NO. This finding fits with previous *in vitro* studies in endothelial cells, in which DCA and CDCA and their taurine conjugates increased intracellular calcium concentration in a dose-dependent manner and therewith NO formation (Nakajima et al., 2000). However, this group later found in rat mesenteric vessels, that glycine conjugates of DCA inhibited the RhoA/Rho-kinase pathway *via* decreased membranous translocation of RhoA in the smooth muscle cell (Jadeja et al., 2018). That is, the effects were due to a reduction in calcium sensitization and not to NO. Previously, this group had shown in rat mesenteric arteries that the glycine-conjugate of DCA inhibits at physiological concentrations, independent of NO, muscarinic receptors, or potassium channels, which confirmed earlier studies in perfused mesentery of the rat and in arterial rings. In this study TDCA > TCDCA > TUDCA induced NO-independent relaxation mainly due to inhibition of calcium entry into the smooth muscle cell (Pak et al., 1994). Last but not least, Dopico et al. (2002) showed in a very elegant patch-clamp study on isolated rabbit mesenteric artery and pulmonary artery smooth muscle cells that unconjugated hydrophobic bile acids increase BK_Ca_ (calcium-activated potassium channel) activity and thus counteract cell contraction.

**Figure 6:**
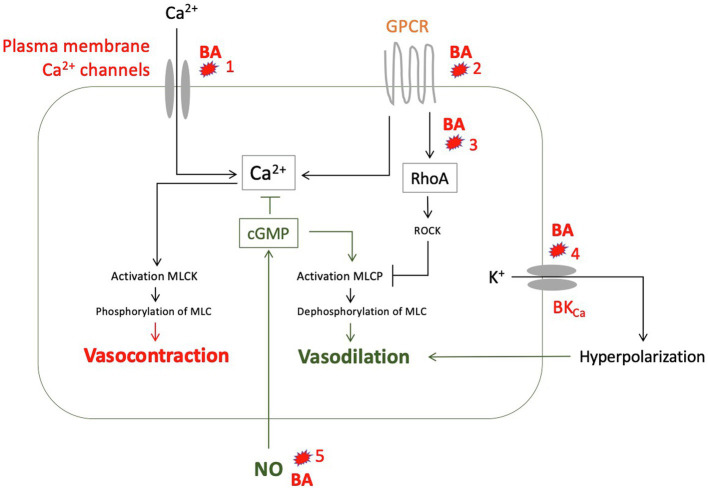
Suggested vasodilatory effects of various bile acids (BA) as to the results of experimental studies: (1) Reduction of calcium influx into smooth muscle cells (Pak et al., 1994). (2) Alteration of G protein-coupled receptors (GPCR), for example by changing membrane fluidity mainly through lipophilic bile acids (Ljubuncic et al., 2000). (3) Inhibition of translocation of RhoA to the cell membrane through glycin conjugates from deoxycholic acid (DCA), thus preventing activation of Rho kinase (ROCK) and ROCK-mediated myosin light chain (MLC) phosphatase inhibition (Jadeja et al., 2018). (4) Activation of calcium-activated potassium channels (BK_Ca_) by hydrophobic bile acids (Dopico et al., 2002). (5) Increased formation of NO by endothelial cells through taurine-deoxycholic acid (DCA) mediated by M3 receptors (Nakajima et al., 2000; Khurana et al., 2005), mediated by TGR5, especially lithocholic acid (LCA), chenodesoxycholic acid (CDCA), and DCA (Guo et al., 2016a), or by activation of farnesoid X receptor (FXR), especially by CDCA (Li et al., 2008). GPCR, G protein-coupled receptor; MLC, myosin light chain; MLCK, MLC kinase; MLCP, MLC phosphatase; and NO, nitric oxide.

In summary, there are a number of *in vitro* studies showing that predominantly hydrophobic bile acids have vasodilatory effects, mostly NO-independent (Figure 6) and at concentrations that can also be found in serum of patients with liver cirrhosis.

## TGR 5 Mediated Effects of Bile Acids on the Vasculature

The G-protein-coupled bile acid receptor Gpbar1 (TGR5) triggers a number of intracellular signaling pathways *via* its natural endogenous ligands, primary and secondary conjugated and unconjugated bile acids (in descending order: LCA > DCA > CDCA > UDCA > CA), including smooth muscle cell relaxation (Guo et al., 2016a,b). For example, bile acids (TLCA and TCDCA) release NO intrahepatically *via* sinusoidal eNOS in the rat *via* TGR5 (Keitel et al., 2007). Expression of TGR5 was also detected in endothelial cells from various human vessels, as was activation/phosphorylation of eNOS by TLCA (Kida et al., 2013). In dogs, TGR 5 agonists cause peripheral vasodilation and reduction of mean arterial blood pressure (NO-independent), most likely due to a cross-talk to BKca, but the mechanism is still unclear (Fryer et al., 2014).

## FXR-Mediated Effects of Bile Acids on the Vasculature

The FXR belongs to the ligand-activated transcription factors. Important activating ligands are conjugated bile acids, especially CDCA > LCA = DCA > CA. In addition, synthetic ligands such as obeticholic acid (OCA) are used for the treatment of cholestatic and metabolic liver diseases. FXR is expressed in the liver and also in the intestine and regulates bile acid uptake and neosynthesis in the liver-gut axis, partly in interaction with TGR5 and FGF19. FXR is also expressed in endothelial cells. Here, activating ligands – such as CDCA – lead to increased expression of eNOS and increased formation of NO (Li et al., 2008) and also to a decrease in endothelin-1 expression (He et al., 2006). Both phenomena suggest the possibility that bile acids, especially CDCA, may also produce vasodilation *via* FXR. Finally, activation of FXR on rat aortic smooth muscle cell leads to increased expression of angiotensin II type 2 receptor, which is also thought to have a vasodilatory function (Zhang et al., 2016).

## Intestine

In healthy individuals, the bile acid pool in the body is distributed mainly through the liver (<1%), intestine (85–90%) and the gallbladder (10–15%; Carey and Duane, 1994; Chiang and Ferrell, 2020). The proportion that reaches the systemic circulation in healthy individuals through spill over is low. As mentioned above, about 20% of bile acids are not actively absorbed from the ileum into the portal blood and enter the colon (Chiang and Ferrell, 2020) where they are deconjugated by bile salt hydrolases (BSH) and then passively absorbed (Begley et al., 2005). Only 5% of total bile acids leave the body *via* feces. In the resident microbiome of the colon, BSH are redundantly available even with a change in the microflora (Ridlon et al., 2015). By contrast, the 7-alpha-dehydroxylation of primary bile acids (CA to DCA and CDCA to LCA) is restricted to the genera *Eubacterium* and *Clostridium* (Firmicutes), *via* the bile acid inducible (*bai*) operon. This means that the conversion to secondary bile acids is more subject to a change in the microbiota (Begley et al., 2005) than deconjugation. In liver cirrhosis, bacterial overgrowth of the upper intestine has been demonstrated in many studies (Gurusamy et al., 2021), i.e., bacterial biotransfomation of bile acids can already take place in the small intestine. In cirrhosis of the liver, it is not only the bacterial overgrowth of the upper intestinal tract that accompanies disease, but also a dysbiosis (Qin et al., 2014; Arab et al., 2018; Fairfield and Schnabl, 2021) that takes place mainly in the large bowel. The major phyla in the colon in healthy individuals are Firmicutes and Bacteroidetes as well as Proteobacteria (Bajaj et al., 2018; Haran and McCormick, 2021). In liver cirrhosis occurs a shift in favor of the Bacteroidetes. The essential taxa containing enzyme for 7-alpha-dehydroxylation, however, belong to the Firmicutes (Ridlon et al., 2015), a possible explanation for the decrease of secondary bile acids in liver cirrhosis. Furthermore, the diversity of the microbiota decreases – also reflected by a reduction in the number and diversity of fecal bacterial genes (Qin et al., 2014). This shift appears to occur earlier and more severely in alcoholic liver damage, particularly in alcoholic hepatitis with underlying cirrhosis. In alcoholic cirrhosis, candida is also found more frequently in the stool, microorganisms increasingly considered to have pathogenic influence (Yang et al., 2017).

Taken together, dysbiosis in cirrhosis favors the accumulation of primary bile acids in the stool. The development of the dysbiosis is often initially caused by food and beverages and is then reinforced or perpetuated by the liver disease. Comprehensive studies to what extent change of intestinal microbiota influences the plasma BA pattern and hemodynamics are still missing. But a link must exist.

## Indirect Effect *Via* Inflammation

With the deterioration of liver function, inflammatory markers (Trebicka et al., 2019), bile acid concentration (Mannes et al., 1986), and bilirubin (Kamath et al., 2001) increase in blood with simultaneous alteration of hemodynamics (Maroto et al., 1994; Trebicka et al., 2011). Unfortunately, the mere association of the quantitative changes of these different parameters does not help us to establish a possible pathogenetic role of bile acids and their interaction with inflammatory markers. Release of cytokines (e.g., TNFa, IL6, and IL8) can lead to vasodilation *via* the endothelium by activation of iNOS and formation of NO. It is also possible that chronic inflammation induces a change in the phenotype of the smooth muscle cell with a decreased response to vasoconstrictors. Finally, there is an indirect link to bile acids, e.g., through their effect on the immune system (Chen et al., 2019). According to *in vitro* studies, bile acids may activate the NLRP3 inflammasome and Il-1ß secretion in macrophages by TGR5/EGFR signaling (Gong et al., 2016). By contrast, others showed that TGR5 activating bile acids reduce the formation of proinflammatory cytokines by monocytes and macrophages *via* the corresponding receptor on these immune cells (Leonhardt et al., 2021). Such effects would on the one hand inhibit an adequate immune response, but on the other hand could attenuate vasodilation *via* cytokines. Furthermore, activation of FXR (which may directly cause vasodilation) supports intestinal barrier function and thus may prevent vasodilating inflammatory stimuli coming from the gut (Inagaki et al., 2006; Verbeke et al., 2015; Fiorucci et al., 2021).

In summary, one could speculate that different bile acids indirectly modulate vascular tone *via* their differential immunomodulatory effects on TGR5 and FXR, respectively. However, we do not know how such effects play out across the body (lymphoid tissue, blood compartment, and liver). Thus, it remains elusive whether and in which direction the change in bile acid concentration and composition in liver cirrhosis indirectly drives vasodilation *via* inflammation.

## Heart

Although not the focus of this paper, the influence of different bile acids on cardiac function and cardiomyocytes cannot be ignored. We refer here to summaries (Bernardi, 2013; Voiosu et al., 2017; Vasavan et al., 2018). Cirrhotic cardiomyopathy is characterized by systolic and diastolic dysfunction as well as by electrophysiological changes (Bernardi, 2013; Voiosu et al., 2017). Cardiac impairment increases with the degree of liver dysfunction. Latent heart failure can rapidly progress to decompensation due to infections, volume loading (e.g. via insertion of TIPS), or surgery. The role played here by various bile acids is unclear. As in the smooth muscle cell, an influence on transmembrane ion channels and on adrenoceptors by altering the fluidity of the cell membrane cannot be ruled out. Cardiomyocytes express TGR5 and respond to FXR ligands. Mice models with elevated bile acids show an alteration of cardiac metabolism and function (Desai et al., 2017). According to *in vitro* studies, analogous to vascular smooth muscle cells, lipophilic bile acids mainly impair cell function, among others by damaging mitochondria. Impairment of cell contraction, ß-adrenoceptor activation, and induction of arrythmias by hydrophobic bile acids were observed in these mostly animal studies (Zavecz and Battarbee, 2010).

## Possible Interventions

Finally, the question that remains is whether it might be useful to modulate the bile acid pool in liver cirrhosis in order to counteract the hyperdynamic circulatory disturbance, a question that has been raised before and by others. The rationale behind is quite evident:

- Cirrhosis of the liver causes hyperdynamic circulatory disturbance with significant extrahepatic consequences.
- In liver cirrhosis, there is a marked change in bile acid metabolism associated with a significant increase in the concentration of bile acids in the systemic circulation.
- The vasodilatory effect of especially hydrophobic bile acids is well-established.

Therefore, it would be interesting to examine whether a modulation of the bile acid pool might counteract the pathophysiology described above. Such an approach could have implications not only for hemodynamics but also for hepatic inflammation and intestinal dysbiosis in patients with liver cirrhosis, given the interaction between bile acids and intestinal bacteria. However, such an attempt deals with extremely complex systems. For example, we do not really know the distribution of individual bile acids (free, taurine or glycine-conjugated) and their concentration in the different organs, the systemic arterial or splanchnic vascular compartments, the amount of binding to albumin and lipoprotein or even their concentration and distribution along the intestine. One could even go so far as to assume, that bile acids act on the cardiovascular system *via* the central nervous system (Kiriyama and Nochi, 2019). All this makes it very difficult to predict the effect of such an intervention. This means that – as Gregorian creatures – we would have to adopt a trial and error approach on the basis of an educated guess (Dennett, 2017).

What could be considered?

- Early modulation of the pool towards individual bile acids that do not lead to vasodilation.
- Influencing the bile acid metabolism *via* TGR 5 or FXR.
- Influencing the bile acids *via* the intestinal flora.

### Altering the Pool by Application of Bile Acids or Other TGR5/FXR Modulators

Two bile acids, even as conjugates, have little vasoactive effect: cholic acid and UDCA (Figure 5). As explained, the cholic acid pool decreases with increasing decompensation of liver cirrhosis. Cholic acid is available as orphan drug for expansion through exogenous supply. But there are no long-term observations in cirrhosis, and one would have to expect that the loss of CA into the intestine would lead to an increase in the occurrence of DCA. DCA, a lipophylic bile acid, is hepatotoxic and has a vasodilatory effect. In contrast, UDCA has been used for many years in liver diseases without harm and even has a proven life-prolonging effect in PBC.

### UDCA Application

Ursodeoxycholic acid is a secondary/tertiary bile acid formed in humans by intestinal microorganisms from CDCA in small amounts. It is contribution to the human bile acid pool is normally very low (1–3%). We have known for about 40 years that UDCA has a “hepatoprotective” effect. There is good evidence for the beneficial effect of UDCA in PBC (Poupon, 2014). Yet, the proof of a beneficial influence for other chronic liver diseases such as PSC or metabolic liver diseases is much lower or nonexisting (Reardon et al., 2016; de Vries and Beuers, 2017). Nevertheless, no studies showed a deterioration of serum liver tests under UDCA; on the contrary, a decrease in serum aminotransferases, alkaline phosphatase, and y-GT was observed in the majority of trials. The accessible data on hemodynamic parameters in these studies are sparse or nonexistent. Noteworthy, in icteric patients with decompensated alcoholic cirrhosis, UDCA administration had an unfavorable effect (Pelletier et al., 2003) as when administered at very high doses (28–30 mg/kg/d) in patients with PSC (Lindor et al., 2009).

Ursodeoxycholic acid is hydrophilic and, as explained above, has hardly any vasoactive properties. According to recent studies its hepatoprotection is caused by influencing intracellular calcium-dependent signals, improvement of the transporter function of the apical membrane of hepatocytes, anti-apototic effects and the establishment of a “bicarbonate umbrella” at the apical cholangiocyte membrane (Beuers et al., 2015). The displacement of hepatotoxic bile acids during long-term, but not short-term treatment could also play a role. However, treatment with UDCA (1 month) in five patients with PBC or PSC did not lead to a reduction in the pool of hydrophobic bile acids DCA or CDCA (Beuers et al., 1992). When looking at the serum levels of patients with PBC before and after 2 years of therapy with UDCA, there is a slight increase in total bile acid concentration in plasma (29 to 31 μM) with a significant decrease in CA and CDCA, constant serum levels of DCA, a slight increase in LCA, and a very significant increase in UDCA (Poupon et al., 1993; Chen et al., 2020). Thus, UDCA treatment increases the ration of hydrophilic/hydrophobic bile acids in serum, which should be favorable concerning vasodilation. Could this – by displacing hydrophobic bile acids in the systemic circulation – have an effect on hemodynamics in the chronic liver disease patient? We do not know for sure. The available data are not rousing.

In healthy subjects, 4 weeks of UDCA vs. placebo does not affect basal or postprandial portal flow or cardiac parameters, such as cardiac output, ejection fraction and QT time. Only diastolic blood pressure was slightly but significantly reduced (Schiedermaier et al., 2000). As known from other studies, UDCA leads to an immediate (Sailer et al., 1996; Schiedermaier et al., 2000; Neubrand et al., 2004) increase in gallbladder volume. The mechanism of this effect on a smooth muscle organ is still unclear; one explanation could be the inhibition of cholinergic pathways (Neubrand et al., 2004). To what extent this effect on the gallbladder influences intestinal metabolism of BA is unclear.

Two decades ago, clinical researchers already tested the hypothesis of influencing the unfavorable hemodynamics in liver cirrhosis by modulating the bile acid pool. In patients who had received TIPS for refractory ascites, treatment with UDCA (15 mg/kg) for 1 month did not affect hemodynamic parameters (systemic, renal, and forearm blood flow), but (even in cirrhotics with ascites without TIPS) resulted in significant sodium retention, which was interpreted as an effect on the proximal tubule (Wong et al., 1999). To our knowledge, such an effect of UDCA on the sodium balance in patients with liver cirrhosis has never been further systematically investigated.

In a small cohort of patients with compensated cirrhosis (PBC, posthepatitic, Child A), 1 month of treatment with UDCA (13 mg/kg/d) did not significantly affect cardiovascular parameters except for a slight reduction in diastolic volume in the PBC patients and a slight reduction in cardiac output in the patients with PBC cirrhosis (Baruch et al., 1999).

There is only rudimentary research on the effect of UDCA on human portal pressure. In the rat model (bile duct ligation), administration of UDCA for 1 month resulted in a decrease in portal venous blood pressure *via* a decrease in intrahepatic resistance, which was interpreted as an intrahepatic antioxidant effect (Yang et al., 2009). In a small series of patients with PBC and discretely elevated portal pressure, portal pressure was unchanged after 2 years of UDCA treatment, while it slightly increased in the placebo patients. Information on other hemodynamic parameters is not available (Huet et al., 2008).

There is evidence from animal experiments – and also in humans (Rainer et al., 2013) – that hydrophobic bile acids have an arrhythmogenic effect and influence cardiac function (see above); on the other hand, UDCA may be cardioprotective (Zavecz and Battarbee, 2010). Small pilot studies in humans additionally indicate that administration of UDCA (and its taurine conjugate) at a dose of 1,000 mg/d for 1 month favorably affect endothelial dysfunction under glucose challenge (Walsh et al., 2016) as well as in coronary artery disease (Sinisalo et al., 1999), and in heart failure (von Haehling et al., 2012). It is unclear how this is mediated. According to animal studies, this effect of UDCA could be induced in a completely different way, namely *via* a reduction of cholesterol crystals in vascular macrophages, where these – *via* stimulation of the inflammasome – have an IL-1-mediated proatherogenic effect and can also cause endothelial dysfunction (Bode et al., 2016).

## Influencing TGR 5 and FXR

Little evidence emerges for TGR5 modulation to affect vasodilation of liver cirrhosis. It may be that free or conjugated CDCA also causes vasodilation in cirrhosis *via* TGR5 and NO liberation (see above and Figure 3) and in the addition *via* inhibition of RhoA/ROCK signaling (Zhu et al., 2020). But the effect of antagonizing TGR5 might counteract favorable antiinflammatory influences mediated *via* TGR5 stimulation (Duboc et al., 2014; Guo et al., 2016b; Deutschmann et al., 2018).

As stated above, FXR stimulation exhibits vasodilator effects *via* several mechanisms. Thus, taurine-conjugated BA mediate NO-fomation *via* the endothelial FXR (Guizoni et al., 2020). Since various steroidal and nonsteroidal (Schwabl et al., 2017) agonists are available, it would be interesting to analyze their effect on hemodynamics in chronic liver disease. In the cirrhotic animal models (rats and mice), feeding with non-steroidal FXR agonists lowered portal pressure by lowering intrahepatic resistance and simultaneously reducing inflammatory stimuli from the gut (Verbeke et al., 2014, 2016). Systemic hemodynamics showed a slight reduction of systemic arterial pressure in rats but not in the mouse model (Schwabl et al., 2017). Similarly, in an animal model of colitis, FXR activation led to downregulation of the expression of pro-inflammatory cytokines and to an improvement of the intestinal barrier (Gadaleta et al., 2011).

The large placebo-controlled study (Nevens et al., 2016) on the effect of the FXR agonist OCA in addition to UDCA in non-cirrhotic patients with PBC incompletely responding to UDCA showed a positive effect on the prognostic surrogate parameters alkaline phosphatase and bilirubin, as well as a reduction in inflammatory serum markers. Detailed analysis of hemodynamic parameters had not been performed in this trial. An increase in cardiovascular adverse events or ECG changes did not occur. This is reassuring considering that cardiotoxicity in animal models is partially FXR mediated (Pu et al., 2013) and that FXR agonists lead to an increase in LDL-cholesterol in serum (Nevens et al., 2016).

In liver cirrhosis – alcoholic and non-alcoholic – and also in alcohol exposure of the pre-cirrhotic patient, there is dysbiosis and concomitant increase of CDCA in serum with reduction of DCA (Brandl et al., 2018; Ciocan et al., 2018). Conjugated and unconjugated CDCA are among the strongest endogenous activators of FXR. Therefore, one could consider blocking – not antagonizing – FXR in patients with liver cirrhosis to counteract putative FXR mediated vasodilation in advanced liver damage. However, this is a double-edged sword, as one would then simultaneously weaken the described anti-inflammatory and hepatoprotective effect of FXR activation. We do not know whether FXR agonists exacerbate hemodynamic circulatory dysfunction in patients with liver cirrhosis.

## Influencing the Intestine

Bile acids influence the microflora in the intestine and, vice versa, bacterial enzymes metabolize bile acids and thus alter the bile acid pool. Interestingly, almost complete reduction of the serum bile acid concentration in animal models (portal vein ligation) with cholestyramine gavage leads neither to change in splanchnic and systemic hemodynamics (Genecin et al., 1990), nor to a reduction of portal pressure. Also, from a theoretical point of view, binding of intestinal bile acids by sequestrants (cholestyramine or colestipol) makes no sense. Total bile acids are already reduced in liver cirrhosis and it would be more important to modulate the composition of individual bile acids with their different direct and indirect effects on intestinal microflora, receptors, vascular smooth muscle cells, or on the heart. Can this be achieved by antibiotics? A small randomized, placebo-controlled trial found improvement in hyperdynamic circulatory dysfunction (Rasaratnam et al., 2003) with 4 weeks of norfloxacin. Rifaximin is a non-absorbable antibiotic with proven effect on hepatic encephalopathy (Bass et al., 2010). While its effect in the intestine has not been fully elucidated, it has been found to reduce the production and absorption of gut-derived toxins and inflammatory stimuli, such as ammonia and endotoxin (Bajaj, 2016). Overall, its effects in the intestine may be more eubiotic than antibiotic (Ponziani et al., 2017). According to uncontrolled trials, rifaximin reduced plasma endotoxin levels and HVPG in alcohol-related decompensated liver cirrhosis (Vlachogiannakos et al., 2009). Furthermore, it lowered the 5-year cumulative probability of decompensation of cirrhosis, including bleeding and encephalopathy, and resulted in better survival (Vlachogiannakos et al., 2013). However, although the data are promising, they are from only one center and as yet remain uncontrolled. A randomized double-blind placebo-controlled trial consisting of a 4-week treatment with rifaximin found no effect on bacterial translocation, HVPG, systemic hemodynamics, kidney function, or vasoactive hormones, including plasma renin (Kimer et al., 2017), and a further study found that there was no short-term effect of rifaximin on systemic inflammatory markers or intestinal bacterial composition (Kimer et al., 2018). This may be due to the technique of sequencing used. A recent study demonstrates significant and profound changes in the microbiota in liver cirrhosis – using metagenomics and not only 16S sequencing – with a favorable effect of rifaximin unlike absorbable antibiotics (Shamsaddini et al., 2021). The extent to which such antibiotic therapies shift individual bile acids in a favorable direction in humans is unclear. Recent studies have demonstrated a reduction of secondary fecal bile acids by rifaximin in mice with humanized stools (Kang et al., 2016). The result of further clinical trials on the role rifaximin on the progress of liver cirrhosis have to be awaited (Caraceni et al., 2021), hopefully with a concomitant focus on bile acid metabolism.

In the intestine – contrary to the vascular system – an increased activation of FXR would be desirable (Flynn et al., 2015) because of its anti-inflammatory effects. But CDCA, the most potent FXR agonist, is already elevated in liver cirrhosis, at least in serum, where it might have vasodilatory effects. Portal pressure reduction might be taken as an indirect indication of a favorable intervention on splanchnic hemodynamics. A meta-analysis found no convincing effect of antibiotics acting in the intestine on portal pressure (Mendoza et al., 2020). Again, it is difficult to distinguish extrahepatic from intrahepatic effects. Relatively specific dysbiosis in therapy-naive patients with PBC was reversed by 6 months of treatment with UDCA (Tang et al., 2018). For all these studies, there are no findings on the change in the bile acid pool, its distribution and associated hemodynamic changes.

Another approach is the modulation of the intestinal microbiota with probiotics, prebiotics or synbiotics (Sarin et al., 2019). Most studies are related to the influence on hepatic encephalopathy in liver cirrhosis of different etiologies. Another study with a mixture of eight probiotic strains found a reduction in hospitalization in predominantly alcoholic cirrhosis, but again, no analyses of the individual bile acids or hemodynamics are available. Plasma renin, aldosterone, and BNP levels decreased significantly in the probiotic group. In the placebo group, there was no change in plasma renin and BNP levels, but a significant increase in aldosterone levels (Dhiman et al., 2014). Any connections to the bile acid pattern and its change as well as relationship to hemodynamic findings remain elusive.

Last but not least, we need more information about diet, liver cirrhosis and its influence on the bile acid pool. Tea, coffee fermented milk products, vegetable and chocolate were associated with a higher diversity with the microbiota in patients with liver cirrhosis, but again there is no data on the relationship of food to bile acids and hemodynamics in these patients (Bajaj et al., 2018).

### Albumin

**Figure 7:**
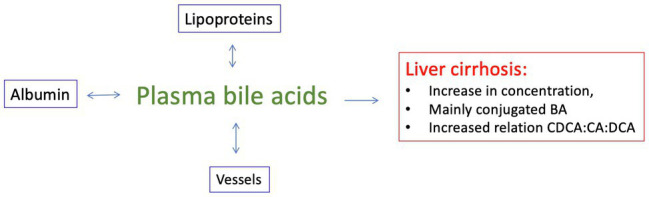
In liver cirrhosis, there are markedly elevated plasma levels of conjugated bile acids in the peripheral circulation, and in alcoholics, especially of CDCA. We know too little about the exchange of these molecules between circulating lipoproteins, plasma albumin and the vascular wall.

Albumin with its oncotic properties is an effective plasma expander. By this, it acts on the baroreceptors and reduces the augmented neurohumoral response in liver cirrhosis. But albumin has further pleiotropic non-oncotic features. Among others, it can bind particles and molecules important for inflammation and it has antioxidant function (Fernandez et al., 2019, 2020; Bernardi et al., 2020; Tufoni et al., 2020). Metabolomic analyses show an increase of a number of molecules – some of intestinal origin – associated with decompensation in liver cirrhosis (Bajaj et al., 2020). This leads to the question whether vasoactive molecules such as bile acids could be bound by the administration of fresh albumin in order to compensate for a reduced albumin concentration and its impaired structural integrity in liver cirrhosis. Unfortunately, there are no studies on the complex question of the exchange of bile acid molecules between exogenously applied and endogenous albumin (Figure 7). There is also a lack of studies on how the various bile acids – conjugated and unconjugated – dissociate from the albumin molecule or the lipoproteins and then exert their effect on the endothelium or the smooth muscle cell. The question could be roughly approached in a first step by determining only the plasma concentration of bile acids before and after album administration.

## Summary and Conclusion

The present work analyzed here on the role of bile acids in the pathogenesis of extrahepatic vasodilation and cardiovascular alteration is not conclusive. There is good evidence that hydrophobic bile acids in particular can lead directly and indirectly to vasodilation. But in liver cirrhosis, the pool of DA, the bile acid with the highest lipophilicity (Roda et al., 1990) actually decreases. On the other hand, the plasma concentration of serum bile acids increases markedly in the systemic vascular compartment, mainly in favor of CDCA and its conjugates. Here, concentrations are theoretically reached for which *in vitro* studies have shown that CDCA can induce vasodilation. But we have no data at all on the exchange of bile acids between albumin or lipoproteins and the vascular endothelium or vascular smooth muscle cell where they could have a direct or indirect vasodilatory effect. Even assuming that CDCA would induce vasodilation in liver cirrhosis on the one hand there remains its beneficial anti-inflammatory effect as an FXR agonist on the other hand. UDCA is hydrophilic, hardly vasoactive and furthermore hardly stimulates TGR5 or FXR. Thus, this bile acid would be the most convenient candidate, an idea suggested early on by other clinical investigators. But the evidence that administration of UDCA could be effective is most limited. Nevertheless, with the exception of PBC, the studies are too short and there is too little experience in the stage of cirrhosis. We lack long-term randomized placebo-controlled long-term trials (including hemodynamic endpoints) in early stage cirrhosis, regardless of etiology.

## Suggestions for Future Research

- Studies on the modulation of the microbiome and its effect on the bile acid pool, serum bile acid pattern and hemodynamic parameters.
- Studies on the distribution of different individual bile acids between albumin and lipoproteins in serum and their dissociation into vascular cells or to their receptors and membranes.
- Controlled studies of early and long-term administration of UDCA – independent of etiology – on liver function and hemodynamic parameters such as cardiac output, heart rhythm, blood pressure and portal pressure in patients with advanced fibrosis or early compensated cirrhosis.
- Studies on the alteration of the serum bile acid pattern after TIPS insertion and including this into parameters for multivariate analysis in relation to hemodynamic changes.

*For Gustav Paumgartner in memory of the Pichlschloss transport meeting in October 2018*.

## Author Contributions

TS wrote the first draft of the manuscript. MH, JT, and UB corrected and reformulated sections of the manuscript. All authors contributed to the article and approved the submitted version.

## Conflict of Interest

The authors declare that the research was conducted in the absence of any commercial or financial relationships that could be construed as a potential conflict of interest.

## Publisher’s Note

All claims expressed in this article are solely those of the authors and do not necessarily represent those of their affiliated organizations, or those of the publisher, the editors and the reviewers. Any product that may be evaluated in this article, or claim that may be made by its manufacturer, is not guaranteed or endorsed by the publisher.

## Figures and Tables

**Table 1:** 

Terms	Definitions
ACE2	Angiotensin-converting enzyme 2
ACLF	Acute on chronic liver failure
BA	Bile acids
BNP	Brain natriuretic peptide
BSH	Bile salt hydrolases
CA	Cholic acid
cAMP	cyclic adenosine monophosphate
CDCA	Chenodeoxycholic acid
cGMP	cyclic guanosine monophosphate
CO	Carbon monoxide
CYP7A1	Cholesterol 7-alpha-hydroxylase
DAG	Diacyl glycerol
DCA	Deoxycholic acid
eNOS	Endothelial nitric oxide synthase
FGF	Fibroblast growth factor
FXR	Nuclear farnesoid X receptor
GDCA	Glycine conjugated DCA
GPBAR1	G protein-coupled bile acid receptor 1 or TGR5
GRK	G protein-coupled receptor kinase
GTP	Guanosine triphosphate
GTPases	Enzymes that hydrolise GTP to GDP (guanosine diphosphate)
HSC	Hepatic stellate cell
HVPG	Hepatic venous pressure gradient (surrogate for portal pressure)
iNOS	Inducible nitric oxide synthase
IP3	Inositol 1,4,5-triphosphate
LCA	Lithocholic acid
L-NAME	N(ω)-nitro-L-arginine methyl ester
MLC	Myosin light chains
NO	Nitiric oxide
NPY	Neuropeptide Y
NOS	Nitric oxide synthase
nNOS	Neuronal nitric oxide synthase
PBC	Primary biliary cholangitis
PSC	Primary sclerosing cholangitis
PLC	Phospholipase C
PKC	Protein kinase C
RAAS	Renin-angiotensin-aldosterone system
RhoA	Ras homolog family member A
ROCK	Rho-kinase
SHP	Small heterodimer partner
TCDCA	Taurine conjugated CDCA
TDCA	Taurine conjugated DCA
TGR5	See GPBAR1
TIPS	Transjugular intrahepatic portosystemic shunt
TUDCA	Taurine conjugate of UDCA
UDCA	Ursodeoxycholic acid
VSCM	Vascular smooth muscle cell
